# Avaliação da implementação de uma estratégia nacional intersetorial de prevenção à obesidade infantil no Brasil

**DOI:** 10.1590/0102-311XPT223624

**Published:** 2026-01-09

**Authors:** Charlise Frasson Pereira, Rubia Carla Formighieri Giordani, Vanessa Daufenback, Risia Cristina Egito de Menezes, Jonas Augusto Cardoso da Silveira

**Affiliations:** 1 Programa de Pós-graduação em Políticas Públicas, Universidade Federal do Paraná, Curitiba, Brasil.; 2 Programa de Pós-graduação em Alimentação e Nutrição, Universidade Federal do Paraná, Curitiba, Brasil.; 3 Faculdade de Nutrição, Universidade Federal de Alagoas, Maceió, Brasil.

**Keywords:** Políticas Públicas, Avaliação de Programas e Projetos de Saúde, Difusão de Inovação, Colaboração Intersetorial, Public Policies, Program Evaluation, Diffusion of Innovation, Intersectorial Collaboration, Políticas Públicas, Evaluación de Programas y Proyectos de Salud, Difusión de Innovaciones, Colaboración Intersectorial

## Abstract

Avaliou-se a implementação do PROTEJA, uma estratégia nacional intersetorial de prevenção e atenção à obesidade infantil direcionada para municípios de pequeno porte. Através de estudo qualitativo de casos múltiplos fundamentado na ciência da implementação, realizaram-se 35 entrevistas com responsáveis técnicos municipais das cinco macrorregiões brasileiras provenientes de diferentes cenários de desenvolvimento humano e de aderência à inovação. A identificação e categorização dos facilitadores e barreiras da implementação foram baseadas no *Consolidated Framework for Implementation Research* (CFIR, Quadro Conceitual Consolidado para Pesquisa de Implementação) por meio de análise de conteúdo. Identificou-se como fatores facilitadores a governança intersetorial, o planejamento e o diagnóstico territorial participativo, o apoio técnico à gestão, a adaptabilidade às realidades locais, a compatibilidade ao rol de serviços da saúde e a percepção de vantagem relativa em relação à programas similares. As principais barreiras foram a dificuldade na utilização dos recursos financeiros, a alta rotatividade e precarização dos responsáveis técnicos, dificuldade em articular agendas para o pleno funcionamento dos espaços de governança, resistência dos profissionais da assistência e baixa prioridade relativa da obesidade infantil como problema público por secretários e prefeitos. Ao articular diretrizes, apoio técnico e incentivos financeiros, viabilizou-se a implementação do PROTEJA. A análise orientada pelo CFIR evidenciou como a interação entre elementos técnicos (instrumentos de planejamento e metas), organizacionais (estruturas e redes intra e intersetoriais) e humanos (capacidades instaladas e trajetórias de aprendizado) facilitaram ou dificultaram a concretização da política.

## Abstract (en)

This study assessed the implementation of PROTEJA, which is a national intersectoral strategy for the prevention and treatment of childhood obesity targeting small municipalities. Through a qualitative multiple-case study grounded in implementation science, 35 interviews were conducted with municipal technical leaders from the five macroregions of Brazil from different human development scenarios and different degrees of adherence to innovation. The identification and categorization of facilitators and barriers with regards to implementation was based on the Consolidated Framework for Implementation Research (CFIR) using content analysis. The facilitators identified were intersectoral governance, participatory territorial planning and diagnosis, technical support for management, adaptability to local situations, compatibility with the list of health services, and the perception of relative advantage over similar programs. The main barriers were difficulty in the use of financial resources, the high turnover of technical leaders, difficulty in coordinating agendas for the full functioning of spaces of governance, resistance from healthcare professionals, and low relative priority given to childhood obesity as a public health problem by municipal authorities. The implementation of PROTEJA was made possible by coordinating guidelines, technical support, and financial incentives. The analysis guided by CFIR revealed how the interaction among technical (planning instruments and goals), organizational (intra- and intersectoral structures and networks), and human (capabilities and learning curves) elements facilitated or hindered the implementation of the policy.

## Abstract (es)

Se evaluó la implementación de PROTEJA, una estrategia nacional intersectorial de prevención y atención a la obesidad infantil dirigida a municipios pequeños en Brasil. A partir de un estudio de caso múltiple cualitativo basado en la ciencia de la implementación, se realizaron 35 entrevistas con gerentes técnicos municipales de las cinco macrorregiones brasileñas de diferentes niveles de desarrollo humano y adhesión a la innovación. La identificación y categorización de los facilitadores y de las barreras de implementación se basó en el *Consolidated Framework for Implementation Research* (CFIR, Marco Consolidado para la Investigación sobre la Implementación) mediante el análisis de contenido. Los factores facilitadores identificados fueron la gobernanza intersectorial, la planificación y el diagnóstico territorial participativo, el apoyo técnico para la gestión, la adaptabilidad a las realidades locales, la compatibilidad con la lista de servicios sanitarios y la percepción de ventaja relativa en relación con programas similares. Las principales barreras fueron la dificultad en el uso de los recursos financieros, la alta rotación y precariedad de los gerentes técnicos, la dificultad para articular agendas para el pleno funcionamiento de los espacios de gobernanza, la resistencia de los profesionales de la atención y la baja prioridad relativa de la obesidad infantil como problema público por parte de secretarios y alcaldes. Al articular lineamientos, apoyo técnico e incentivos financieros, se hizo posible la implementación de PROTEJA. El análisis guiado por el CFIR mostró cómo la interacción entre aspectos técnicos (instrumentos y metas de planificación), organizacionales (estructuras y redes intrasectoriales e intersectoriales) y humanos (capacidades instaladas y trayectorias de aprendizaje) facilitó u obstaculizó la implementación de la política.

## Introdução

Entre 2020 e 2035, a prevalência de obesidade em crianças e adolescentes nas Américas deve crescer de 20% para 33% em meninos e de 16% para 26% em meninas. No Brasil, estima-se um acréscimo anual de 4,4% ^1^. A obesidade infantil causa impactos biopsicossociais e altos custos aos sistemas de saúde ^2^ ^,^ ^3^ ^,^ ^4^ ^,^ ^5^, refletindo um problema público cuja reversão exige mudanças estruturais entre Estado e sociedade ^6^. 

A Década de Ação da Nutrição (2016-2025) da Organização das Nações Unidas (ONU) impulsionou o tema na agenda brasileira com o Plano de Ações Estratégicas para o Enfrentamento das Doenças Crônicas e Agravos Não Transmissíveis (2021-2030) ^7^ ^,^ ^8^. Neste contexto, a Coordenação-Geral de Alimentação e Nutrição (CGAN) do Ministério da Saúde instituiu a Estratégia Nacional para Prevenção e Atenção à Obesidade Infantil (PROTEJA) ^9^, voltada à reorganização do cuidado e à promoção de ambientes saudáveis. 

PROTEJA é o acrônimo dos eixos estruturantes da estratégia (Primeiro Contato, Responsabilização, Organização, Transformação, Educação, Janela de Oportunidade e Ambientes); é composta por 20 ações essenciais (obrigatórias) e 41 complementares (os municípios deveriam pactuar, no mínimo, cinco ações no momento da adesão) ^10^. Foram elegíveis para o incentivo financeiro (R$ 14,51 por criança menor de 10 anos por ano, por três anos) ^11^ municípios com até 30 mil habitantes que, em 2019, apresentaram prevalência de excesso de peso ≥ 15%, cobertura de estado nutricional ≥ 50% e algum registro de marcadores de consumo alimentar no Sistema de Vigilância Alimentar e Nutricional (SISVAN) entre menores de 10 anos ^12^. 

Tratou-se de uma política pública setorial (saúde) com fomento à articulação intersetorial ^12^ em âmbito local (microimplementação) ^13^, na qual as Secretarias Municipais de Saúde (SMS) indicaram um responsável técnico (RT; nível operacional) ^14^ para atuar diretamente na gestão da implementação. Entretanto, por se tratar de municípios de pequeno porte, com número limitado de profissionais, notou-se que os RTs também desempenhavam funções assistenciais esporádicas ou acumulavam ambas as atribuições (situações em que o vínculo primário era assistencial). Assim, ao atuar diretamente com a população na realização das ações e deter poder discricionário de decisão e rotinas para a efetivação da política, o RT foi considerado como burocrata de nível de rua (BNR) ^15^. Secretários de saúde foram considerados burocratas de médio escalão (BME), com função gestora de apoio estrutural às atividades operacionais de saúde, e os prefeitos, burocratas de alto escalão (BAE), responsáveis pelo planejamento estratégico (nível institucional) ^14^ ^,^ ^16^ ^,^ ^17^. 

Além do financiamento, os municípios receberam suporte técnico externo remoto de apoiadores regionais (ApR) e locais (ApL), que atuaram em conjuntos de municípios, organizados por estado, na condução de oficinas formativas bimestrais, eventos de compartilhamento de experiências e suporte individualizado (reuniões mensais ou conforme demanda) ^12^ ^,^ ^18^. Esse desenho de política rendeu ao PROTEJA o prêmio *2022 UN Inter-Agency Task Force and the WHO Special Programme on Primary Health Care Awards* ^19^. 

A implementação de políticas públicas é um fenômeno social influenciado por interpretações e comportamentos dos atores ^20^. Assim, a partir do reconhecimento e compreensão dos contextos coletivos da gestão vivenciados pelos BNR, revelados nas significações dos discursos dos sujeitos ^21^ ^,^ ^22^, pode-se identificar aspectos relevantes para o aprimoramento de políticas, especialmente em municípios de pequeno porte. 

Com base na Ciência da Implementação ^13^ ^,^ ^23^, que visa entender como inovações ^24^ − ou seja, o objeto de interesse a ser implementado − se comportam em contextos reais, este estudo buscou avaliar a microimplementação do PROTEJA, identificando os facilitadores e as barreiras neste processo. 

## Métodos

Trata-se de um estudo qualitativo de casos múltiplos, integrante de um projeto maior de avaliação de impacto e de implementação do PROTEJA a partir de métodos mistos. Para incorporar a pluralidade de experiências, a amostra foi delineada proporcionalmente às adesões por macrorregião, considerando o desempenho nas metas vinculadas aos repasses financeiros e Índice de Desenvolvimento Humano (IDH). Considerando tais critérios, a capacidade operacional da equipe e o número mínimo de três municípios por macrorregião, estimou-se uma amostra 42 municípios (Norte: 11,3%; Nordeste: 41,4%; Centro-oeste: 6,2%; Sudeste: 30,9%; Sul: 11,1%). 

Foram elegíveis para a amostra municípios com RTs designados até o primeiro trimestre/2022. Estes foram escolhidos para as entrevistas por serem os responsáveis institucionais pela implementação da inovação. Os convites foram enviados por e-mail e aplicativo de mensagens. 

As entrevistas ocorreram por videochamadas, entre dezembro/2023 e março/2024, guiadas por um roteiro semiestruturado com 60 perguntas baseadas no *Consolidated Framework for Implementation Science* (CFIR, Quadro Conceitual Consolidado para Pesquisa de Implementação) ^24^, organizadas em 5 domínios e 18 categorias ^25^. Dado o objetivo do estudo, utilizaram-se os quatro domínios do CFIR ^24^ (Quadro 1), relacionados às características da instituição que implementa a Inovação. A perspectiva dos indivíduos sobre o Processo de Implementação (dimensão excluída da análise) será desenvolvida em futura publicação. 

**Quadro 1:** Domínios e categorias do *Consolidated Framework for Implementation Research* (CFIR), aplicados na avaliação da implementação da Estratégia Nacional para Prevenção e Atenção à Obesidade Infantil (PROTEJA).

DOMÍNIO: INOVAÇÃO Estratégia intersetorial para prevenção e atenção à obesidade infantil (PROTEJA), em âmbito local de municípios de pequeno porte, composto por: Núcleo fixo − ações essenciais de vigilância alimentar e nutricional; atendimentos individuais para obesidade em crianças menores de 10 anos; diagnóstico e planejamento territorial. Núcleo adaptável − ações complementares; elementos, estruturas e sistemas adaptáveis da inovação e dos setores envolvidos na implementação do PROTEJA	
Categorias	Definição
Vantagem Relativa	Vantagem de implementar a inovação em relação a outras inovações ou práticas atuais disponíveis
Complexidade	Complexidade do número de etapas (duração, escopo e/ou natureza) necessárias para a implementação
Adaptabilidade	A inovação pode ser adaptada, modificada ou reinventada para atender às necessidades locais
DOMÍNIO: AMBIENTE EXTERNO Setores, organizações e/ou indivíduos externos à SMS − ambiente interno. Trata das relações intersetoriais necessárias para a concretização da inovação que estão fora da gerência do setor saúde	
Categorias	Definição
Rede Intersetorial	Parcerias e conexões que o ambiente interno (responsável técnico e/ou SMS) tem com as organizações externas
Políticas e Leis	Estratégia para difundir a inovação, como: legislações, regulamentos, diretrizes, recomendações, avaliação por desempenho ou outros
DOMÍNIO: AMBIENTE INTERNO Ambiente/instituição onde a inovação é gerida (SMS), desconsiderando as divisões hierárquicas internas do setor. Dividido em características prévias à inovação (Estruturais) e características após a Inovação (Específicas)	
Categorias	Definição
Características Estruturais	Características da estrutura geral persistente à SMS
Rede Intrassetorial	São as parcerias, relacionamentos, formais ou informais, dentro das fronteiras do ambiente interno (SMS)
Recursos Disponíveis	Profissional: forma de contratação, estruturas de trabalho (responsabilidades, horários, turnos e plantões, ordem e arranjo de tarefas, procedimentos de trabalho e gerenciamento de cargas de trabalho, autonomia e/ou centralização de decisão, definição de funções); Financeiro; Espaço físico, equipamentos, estado de conservação e qualidade; Tecnologia da Informação
Comunicações	Práticas formais e informais de compartilhamento de informações dentro das fronteiras do ambiente interno (SMS)
Características Específicas	Características adquiridas após a adesão a inovação, sendo necessárias e diretas para a sua implementação
Tensão pela Mudança	A situação atual era intolerável ou necessitava de mudança
Prioridade Relativa	Importância dada à implementação da inovação dentro da organização
Compatibilidade	Adaptação da inovação aos fluxos e sistemas de trabalho
Acesso ao Conhecimento e Informação	Facilidade de acesso a informações, orientação e/ou treinamento para a implementação da inovação. São acessíveis, compreensíveis e possíveis de incorporá-los nas tarefas de trabalho
DOMÍNIO: PROCESSOS DE TRABALHO DE IMPLEMENTAÇÃO Atividades e estratégias que foram utilizadas para implementar a inovação	
Categorias	Definição
Planejamento	Grau em que processos, fluxos ou tarefas voltadas para a implementação de uma intervenção são desenvolvidos antecipadamente e a qualidade dos métodos adotados
Execução	Cumulatividade dos passos para a implementação da inovação de acordo com o plano de ação elaborado
Monitoramento/Avaliação	*Feedback* quantitativo e qualitativo sobre o progresso e a qualidade da implementação, acompanhado de relatórios regulares pessoais e de equipe sobre o progresso e a experiência

SMS: Secretaria Municipal de Saúde.

Fonte: adaptado de Damschroder et al. ^24^.

Como pré-teste, o roteiro foi aplicado em uma RT que não faria parte da pesquisa, para verificar a adequação à população-alvo. Posteriormente, foram realizados ajustes na escrita e na ordem das perguntas, com base na experiência do piloto e nas discussões do grupo de pesquisa.

**Figura 1:**
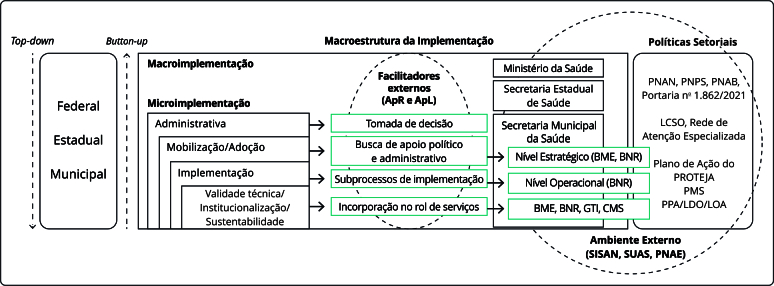
Modelo lógico da macroestrutura de implementação da Estratégia Nacional para Prevenção e Atenção à Obesidade Infantil (PROTEJA).

O CFIR é uma estrutura conceitual com taxonomia padronizada para pesquisas de implementação, aplicado em indivíduos com influência sobre a adoção, implementação e sustentabilidade da Inovação (desfechos da implementação) ^24^. A Figura 1 apresenta o modelo lógico para compreensão dos processos e desfechos de implementação do PROTEJA, considerando seu modelo de gestão híbrido ^26^ (*top-down*: instituição da estratégia pelo Ministério da Saúde; *bottom-up*: adaptações que redesenharam a política em nível local e regional) e os arranjos setoriais (saúde) e extrasetoriais ^25^. 

ApR: Apoiadores Regionais; ApL: Apoiadores Locais; BME: Burocratas de Médio Escalão; BNR: Burocratas de Nível de Rua; CMS: Conselho Municipal de Saúde; GTI: Grupo de Trabalho Intersetorial; LCSO: Linha de Cuidado do Sobrepeso e Obesidade; LDO: Lei de Diretrizes Orçamentárias; LOA: Lei Orçamentária Anual; PNAN: Política Nacional de Alimentação e Nutrição; PMS: Plano Municipal de Saúde; PNAB: Política Nacional de Atenção Básica; PNAE: Programa Nacional de Alimentação Escolar; PNPS: Política Nacional de Promoção da Saúde; PPA: Plano Plurianual; SISAN: Sistema Nacional de Segurança Alimentar e Nutricional; SUAS: Sistema Único de Assistência Social. Fonte: Pereira ^25^.

As entrevistas resultaram no corpus de 35 documentos, os quais foram identificados por códigos alfanuméricos e submetidos à análise de conteúdo ^27^. Primeiramente, fez-se a leitura flutuante das transcrições, gerando as primeiras intuições sobre seu conteúdo. Em seguida, deu-se a etapa exploratória, na qual os trechos relevantes das falas foram codificados dedutivamente segundo as categorias do CFIR por duas pesquisadoras experientes em métodos e softwares qualitativos. Assim, como trechos polissêmicos foram codificados em mais do que uma categoria/domínio, múltiplos trechos sobre uma mesma categoria foram quantificados segundo a frequência de aparições.

Após codificação, os autores analisaram e interpretaram os dados em função da significação que emergiu do contexto, da literatura e do modelo conceitual do CFIR ^24^ ^,^ ^28^ ^,^ ^29^. Os discursos dos BNR foram unidades de contexto para a compreensão da unidade de registro (recortes das experiências locais). As análises se basearam em uma matriz código-documento ^29^, verificando associações entre temas (códigos) e conteúdos (*corpus*), para gerar tendências e distribuições temáticas.

Os dados foram organizados e quantificados ^29^ ^,^ ^30^ no software ATLAS.ti v. 24 (http://atlasti.com/). A pesquisa foi aprovada pelo Comitê de Ética em Pesquisa com Seres Humanos da Universidade Federal de Alagoas (parecer nº 6.135.172). Todos os entrevistados assinaram o Termo de Consentimento Livre e Esclarecido.

## Resultados 

Trinta e cinco RTs foram entrevistados, sendo a maioria do sexo feminino, nutricionistas e com seis anos ou mais de formação. A maior proporção dos BNR atuava no município há menos de 5 anos, tinha experiência em funções de coordenação e, durante a execução do PROTEJA, também atuava na assistência (Tabela 1). 

**Tabela 1:** Características dos responsáveis técnicos municipais pela implementação da Estratégia Nacional para Prevenção e Atenção à Obesidade Infantil (PROTEJA).

Características da amostra	n	%
Macrorregião		
Norte	5	14,3
Nordeste	11	31,4
Centro-oeste	4	11,4
Sudeste	8	22,9
Sul	7	20,0
Índice de Desenvolvimento Humano		
Alto	8	22,9
Médio	10	28,6
Baixo	16	45,7
Muito baixo	1	2,9
Profissão		
Nutricionistas	21	60,0
Enfermeiros	8	22,9
Fisioterapeutas	4	11,4
Outras	2	5,7
Anos de formação		
1-5	3	8,6
6-10	12	34,3
11-15	13	37,1
16-20	4	11,4
> 21	3	8,6
Sexo		
Feminino	32	91,4
Masculino	3	8,6
Atuação no município (anos)		
1-5	16	45,7
6-10	9	25,7
11-15	4	11,4
16-20	5	14,3
> 21	1	2,9
Experiência dos RTs em funções de coordenação *		
Sim	21	60,0
Não	14	40,0
Atribuições dos RTs nos municípios **		
Coordenação do PROTEJA e de outros programas	11	31,0
Coordenação do PROTEJA e assistência	24	69,0

RT: responsável técnico.

* Referente às experiências de coordenação em políticas, programas ou estratégias anteriores à atuação na gestão no PROTEJA;

** Perfil de atividades dos RT no município (nenhum dos entrevistados atuavam exclusivamente no PROTEJA).

Por meio da análise de conteúdo, foram identificadas 1.623 citações referentes às categorias/domínios do CFIR, sendo 74% referentes a fatores facilitadores do processo de implementação (Tabela 2). A síntese dos facilitadores e barreiras estão disponíveis no Quadro 2. A descrição dos resultados foi organizada pelos domínios, seguido das respectivas categorias agrupadas em facilitadores e barreiras.

**Tabela 2:** Frequência das citações sobre facilitadores e barreiras do processo de implementação da Estratégia Nacional para Prevenção e Atenção à Obesidade Infantil (PROTEJA), entre 2021 e 2023, segundo domínios e categorias do *Consolidated Framework for Implementation Research* (CFIR).

Análise entre código-documento	Facilitadores		Barreiras		Frequência de citações	
	n	%	n	%	n	% *
Domínio/Categorias						
Inovação						
Vantagem Relativa	73	82,9	15	17,1	88	5,4
Complexidade	28	66,7	14	33,3	42	2,6
Adaptabilidade	70	80,5	17	19,5	87	5,4
Ambiente Externo						
Rede Intersetorial	196	81,3	45	18,7	241	14,8
Políticas e Leis	26	78,8	7	21,2	33	2,0
Ambiente Interno						
Rede Intrassetorial	95	74,8	32	25,2	127	7,8
Recursos Disponíveis	91	65,0	49	35,0	140	8,6
Comunicações	71	65,1	38	34,9	109	6,7
Tensão pela Mudança	92	73,6	33	26,4	125	7,7
Prioridade Relativa	79	71,8	31	28,2	110	6,8
Compatibilidade	125	65,8	65	34,2	190	11,7
Acesso ao Conhecimento e Informação	92	85,2	16	14,8	108	6,7
Processos de Trabalho de Implementação						
Planejamento	90	91,8	8	8,2	98	6,0
Execução	57	54,8	47	45,2	104	6,4
Monitoramento/Avaliação	15	71,4	6	28,6	21	1,3
Totais	1.200	73,9	423	26,1	1.623	100,0

* Relativo ao total de citações.

**Quadro 2:** Síntese dos facilitadores e barreiras identificados na implementação da Estratégia Nacional para Prevenção e Atenção à Obesidade Infantil (PROTEJA), entre 2021 e 2023, por meio do *Consolidated Framework for Implementation Research* (CFIR).

FATOR FACILITADOR (Elementos positivos)	BARREIRA (Elementos Negativos)
DOMÍNIO: INOVAÇÃO	
Vantagem Relativa	
Promoção da intersetorialidade Diagnóstico e monitoramento da situação alimentação e nutrição no território Apoio técnico externo Percepção do problema público	Dificuldade para a utilização dos recursos financeiros
Complexidade	
Implementação guiadas por etapas	Muitas etapas para serem realizadas individualmente
Adaptabilidade	
Ampliação do acesso dos usuários aos serviços da saúde por: (a) atendimentos individuais em ambiente alternativo (escola e centros comunitários); (b) oferta de serviços em horário alternativo (noturno) Auxiliou na promoção da alimentação e nutrição, Saúde e atividade física Discricionariedade do BNR	Agenda dos profissionais da saúde e horários de atendimento nas UBS Pouco tempo disponível para atuação com o PROTEJA Sobrecarga de serviços dos profissionais da saúde
DOMÍNIO: AMBIENTE EXTERNO	
Rede Intersetorial	
Estratégia de gestão integrada com foco no território Organização logística das ações	Comunicação ou disponibilidade de tempo dos atores convidados para o GTI Baixos quantitativos de profissionais ou equipes Ausência de uma “norma” local
Políticas e Leis	
Aprovação do plano de trabalho do PROTEJA pelo CMS Incorporação do PROTEJA nos Planos Municipais de Saúde	Ausência da divulgação das informações sobre a implementação Ausência de apresentação do plano de trabalho do PROTEJA para o CMS
DOMÍNIO: AMBIENTE INTERNO	
Rede Intrassetorial	
Cooperação e interação da equipe de saúde Organização dos serviços da SMS Construção rede horizontal, informal e adaptável	Resistência para modificar a forma de execução de tarefas rotineiras para atender novas demandas Baixo engajamento do(a) gestor(a) da saúde
Recursos Disponíveis	
Financeiros: contratação de profissional, aquisição de materiais e equipamentos Infraestrutura: adaptabilidade do local de atendimento e rotinas de trabalho	Humanos: (a) não organização das tarefas, agenda, horário da equipe; (b) defasagem de profissionais da saúde ou a carga horária incompatível com a quantidade de serviços; (c) tecnologia da Informação: baixa conectividade, qualidade dos equipamentos
Comunicações	
Comunicação informal (pessoal ou mensagens eletrônicas) Transferência da informação para as equipes da saúde	Não transferência da informação pela gestão anterior Falha da comunicação intraorganizacional
Tensão pela Mudança	
Percepção do aumento da prevalência da obesidade no território (antes da implementação) Percepção da necessidade de organização da rede de atenção à obesidade infantil	Não percepção da condição obesidade Inviabilidade da oferta do serviço de saúde pela ausência de profissionais da saúde (nutricionista ou preparador físico)
Prioridade Relativa	
BNR: reconhecimento e prioridade ao problema público Divulgação dos dados Compreensão do PROTEJA como extensão do setor saúde, sendo compatível as funções e rotinas de trabalho já existentes Gestor de saúde participativo	Secretarias municipais e/ou gestores da saúde não priorizaram o problema público
Compatibilidade	
Adaptação dos processos e rotinas de atividades do PROTEJA aos fluxos e sistemas de trabalho da SMS Vinculação das atividades a metas obrigatórias e repasse de recursos financeiros Produção de informação (dados de diagnóstico e monitoramento da situação alimentação e nutrição no território)	Resistência dos profissionais de incorporar: (a) rotina do marcador de consumo alimentar e da indicação condição de obesidade; (b) tarefas e fluxos de trabalho
Acesso ao Conhecimento e Informação	
Apoio técnico externo Informações compreensíveis (oficinas de capacitação) Comparação entre pares	Tempo indisponível para estudar a implementação Dificuldades técnicas para acompanhar as oficinas de implementação e as reuniões com as apoiadoras locais Horários das oficinas (comercial) Baixo conhecimento dos representantes das GRS (na fase inicial de implementação)
DOMÍNIO: PROCESSOS DE TRABALHO DE IMPLEMENTAÇÃO	
Planejamento	
Participação do BNR junto ao gestor de saúde para análise e planejamento das ações Elaboração do plano de trabalho no prazo estipulado Metas vinculadas às atividades rotineiras de trabalho	Atraso do prazo estipulado para elaboração ou não execução do plano de trabalho do PROTEJA
Execução	
Construção/ampliação de rede intrasetorial e intersetorial, especialmente com a educação Engajamento dos pais no cuidado Metas e plano de trabalho Locais adequados e lúdicos para as atividades com o público infantil	Dificuldade de acesso das crianças das zonas rurais às UBS Agenda das escolas (intersetorialidade) Agenda dos profissionais da saúde (intrassetorial) Aceitação dos pais da necessidade do cuidado
Monitoramento e Avaliação	
Acompanhamento dos processos de trabalho de implementação Percepção do erro de execução e correção da implementação Discricionariedade do BNR	Ausência de monitoramento e avaliação interno Ausência de correção durante curso de implementação

BNR: Burocrata de Nível de Rua; CMS: Conselho Municipal de Saúde; GRS: Gerências Regionais de Saúde; GTI: Grupo de Trabalho Intersetorial; SMS: Secretaria Municipal de Saúde; UBS: unidade básica de saúde.

### Inovação

#### Facilitadores

Para 83% das citações, houve Vantagem Relativa em implementar o PROTEJA em relação à programas similares, como Programa Saúde na Escola (PSE) e Programa Crescer Saudável, devido à criação do grupo de trabalho intersetorial (GTI), instituição do apoio técnico e da necessidade de realização do diagnóstico da situação alimentar e nutricional, mapeamento dos espaços para prática de atividades físicas e planejamento das ações (P13). 

“*Pra mim o PROTEJA foi o melhor programa que existiu, porque foi o primeiro momento que eu tive a oportunidade de ver os meus indicadores, que a gente não tem tempo de parar de fazer. A gente coleta o dado e lança, mas parar, fazer mapeamento, conhecimento de território. Foi o PROTEJA, que me oportunizou* (...). *Porque foi onde eu pude identificar o bairro que eu tinha maior número de obesidade, foi onde eu pude identificar a escola, para desenvolver as ações nos locais certos* (...). *As etapas elas são necessárias para você ter ali as ações para ser desenvolvido, para você conquistar, chegar no que foi estabelecido. Elas parecem ser muitas, mas quando você começa a colocar toda, cada etapa, ela é muito automática de se realizar e seguir um fluxo natural*” (P13, Sudeste).

Apesar de ter sido uma estratégia complexa, as atividades formativas ligadas ao PROTEJA reduziram a percepção da dificuldade no decorrer da implementação (66,7%), como relata o P13. A flexibilidade para adaptações à realidade local (80,5%) também foi considerada um facilitador para a implementação (P17). 

“*É, é, a gente tem uma parceria intersetorial muito boa com as escolas. Eles cederam o espaço, né? E a gente montou um consultório lá, levou o computador para fazer, está fazendo os marcadores, né? E levou balança pra técnica de enfermagem tá fazendo a antropometria das crianças e o consultório médico é separado da sala de triagem*” (P17, Sudeste).

#### Barreiras

No escopo da Vantagem Relativa, as dificuldades para acessar os recursos financeiros representaram uma barreira para a execução das ações pactuadas. 

“*...uma desvantagem que eu vi do PROTEJA, foi em relação aos recursos, como utilizá-los*” (P24, Nordeste).

“*Mas a desvantagem é só a gente não conseguir usar o dinheiro no lugar certo.* (...) *Tanto que a gente fez várias reuniões já, mas ainda temos todo o dinheiro pra gastar, sabe? Porque pela questão de licitações, a gente não consegue gastar esse dinheiro, aqui no município eles fizeram adesão da nova lei de licitações e a gente não consegue gastar esse valor sem ter licitação. É muito burocrático*” (P26, Sul).

Diante da Complexidade do PROTEJA, a carência de apoio institucional foi uma barreira para a concretização das ações, deixando os BNR isolados na implementação (P18). 

“*Não, eu achei que ia ser tranquilo, né? Ah se ele deu para nutricionista fazer, eu dou conta, eu sozinha eu vou dar conta. Aí na hora que você começa a estudar e vê que tem prazo pra entregar, que tem etapa pra ser cumprida, você começa a ver que realmente você sozinha não dá conta. Aí você começa a falar que não dá conta e pedir ajuda pra dar continuidade, ninguém está nem aí*” (P18, Sudeste).

Independentemente do apoio setorial, a indisponibilidade de tempo e sobrecarga de serviços para os profissionais pesou sobre qualidade das ações realizadas (P10).

“*Ele não tinha muito tempo pra fazer as atividades física com as crianças, geralmente reuniu uma vez por mês e isso você sabe que não resolve. Então não foi muito efetivo pra gente o PROTEJA*” (P10, Centro-oeste).

### Ambiente Externo

#### Facilitadores

A categoria com maior número de citações foi a Rede Intersetorial, na qual 81,3% das citações apontaram que a construção do GTI e as interações entre os membros conferiu estabilidade e organização logística para a execução das ações que estavam fora das competências da SMS (P29). 

“*Saúde e educação* (...) *Secretaria de Obras se envolveu bastante e na questão da Secretaria do Meio Ambiente a Vigilância Sanitária e a Emater* (...) *Meio Ambiente e Agricultura que a gente foi visitar as hortas, os produtores da região né?*” (P29, Sul).

Além disso, a formação destas redes reduziu a alienação dos gestores em relação às agendas e processos de trabalhos de seus pares nos outros setores (P8).

“*E aí, quem não conhecia o trabalho do outro acaba conhecendo e a gente também percebeu, de fato que não se faz nada sozinho. Eu preciso de alguém que precisa de outro que precisa de outro, e isso é uma rede, né*” (P8, Nordeste).

Esses trechos representam a congregação de diferentes agentes para o diagnóstico situacional, mapeamento dos espaços de promoção da saúde e planejamento das ações, possibilitando a construção de uma estratégia de gestão integrada, com foco nos territórios e fortalecendo o processo de implementação. 

#### Barreiras

Dificuldades de comunicação entre setores e a indisponibilidade de tempo dos atores externos para participar do GTI foram as principais barreiras para o planejamento e concretização das ações. 

“*Às vezes eu me reuni só com a educação. Só com uma Secretaria, porque eu não conseguia reunir todo mundo, porque nem sempre todo mundo estava disponível naquele horário*” (P8, Nordeste). 

“*A gente foi até esse grupo de trabalho. A gente quando organizou em realizá-lo era junto ao PSE, né? Que também faz necessário e já colocaríamos o PROTEJA, mas a gente não teve um sucesso porque o membro da educação nem na reunião que a gente marcou compareceu. E foram três tentativas que a gente realizou*” (P9, Centro-oeste).

### Ambiente Interno

#### Facilitadores

Em relação às características estruturais, práticas de cooperação entre equipes e a presença de redes horizontais (Rede Intrassetorial), permitindo algum grau de informalidade nas relações (Comunicações) − inclusive, mediadas por aplicativos de mensagens −, facilitaram a implementação da inovação. Além disso, a disponibilidade de recursos financeiros e de infraestrutura nas SMS foram fatores importantes para a concretização das ações.

Quanto às específicas, o reconhecimento do problema público da obesidade infantil na categoria Tensão pela Mudança (73,6%) e da priorização pelos BNR enquanto foco de ação (Prioridade Relativa; 71,8%) facilitou a implementação.

A Compatibilidade do PROTEJA foi a categoria com mais citações neste domínio (n = 190). O alinhamento de seu desenho com práticas já realizadas nas SMS possibilitou que os novos processos e rotinas propostos pela inovação pudessem ser facilmente incorporados. Ainda, a coerência com outros programas de saúde (maximização da eficiência) e a vinculação dos repasses financeiros ao cumprimento de metas impulsionou a aceitação e o engajamento ao PROTEJA pelos gestores.

“*Eu comecei a fazer as atividades, mas a equipe foi apoiar depois que o projeto já tava andando, depois de uns 3 a 4 meses começaram a apoiar porque a gente começou a cumprir algumas metas, indicadores de outros programas através do PROTEJA, então aí eles viram que era uma coisa que a gente poderia aproveitar juntos também*” (P15, Sudeste).

As estratégias formativas para os BNR conduzidas pelos apoiadores foram percebidas como um importante fator facilitador para a implementação, uma vez que o Acesso ao Conhecimento e Informação foi a segunda categoria com o maior percentual de citações como fator facilitador (85,2%). 

#### Barreiras

As barreiras mais frequentes estiveram ligadas às categorias Recursos Disponíveis (35%) e Comunicações (34,9%). Devido à alta rotatividade dos RT, somada aos vínculos empregatícios precarizados e documentação insuficiente dos processos de trabalho, criaram-se lacunas de informação entre gestões, muitas vezes, obrigando o novo RT a reiniciar o trabalho. Como o apoio à implementação ocorria, a partir de ferramentas virtuais, a precariedade da infraestrutura de comunicação (equipamentos e internet) em diversos municípios, especialmente na Região Norte; o que reverberou sobre o Acesso ao Conhecimento e Informação, comprometendo a participação síncrona dos BNR nas oficinas e reuniões de apoio técnico. 

As barreiras ligadas à compatibilidade (34%) estiveram ligadas à resistência dos profissionais da assistência em incorporar “novas” rotinas em suas jornadas de trabalho. Na perspectiva da gestão, 28,2% das citações referiram como barreira o não reconhecimento da problemática ligada à obesidade infantil pelos secretários ou prefeitos, reduzindo a Prioridade Relativa da inovação. 

“...*pessoas com cargo acima de mim, que eles não dão uma importância, um olhar pra determinada situação, a importância da prevenção da obesidade infantil, porque é uma criança obesa, provavelmente é um adolescente, um adulto ou idoso, obeso. E a gente sabe que a obesidade ela é uma porta de entrada pra outras doenças*” (P3, Norte).

## Processos de Trabalho de Implementação

### Facilitadores

Para o Planejamento (91,8%), a participação do BNR na elaboração do plano de ação facilitou a execução das ações pactuadas (P24). 

“*Nós sentamos, eu e a secretária, nós chamamos também a secretária de Educação. Aí sentamos para ver o que era aqui, porque lá tinha as ações que eram essenciais. Tinham as outras, e aí a gente foi vendo que já tinha no município e que não tinha e a gente escolheu, nós juntos*” (P24, Nordeste).

No campo da Execução (54,8%), a rede de apoio intersetorial, a explicitação das metas dentro dos planos de ação, a disponibilidade de espaços de promoção da saúde e o engajamento dos pais no cuidado facilitaram a implementação. 

### Barreiras

A principal barreira no Planejamento foi o atraso para a elaboração dos planos de ação (P1). Dos 35 participantes, 27 souberam descrever quando o plano foi elaborado (2021: 11%; 2022: 63%; 2023: 26%); os demais não recordaram.

“[A finalização do plano de ação] *Foi bem no finalzinho do ano* [2023] *e aí a gente...a coordenadora de atenção conversou né com a secretária de Educação só que estava muito, o calendário deles estava muito apertado, estava muito em cima pra gente colocar em prática né então meio que ficou pra esse ano* [2024] *a gente começar a colocar em prática o plano de ação*. (...) *as ações ainda falta a gente colocar em prática, só temos o plano de ação*” (P1, Norte).

Quanto às barreiras da Execução (45%), houve um “espelhamento” dos fatores facilitadores, como a dificuldade de articulação das agendas e dos objetivos institucionais do setor, e entre setores (P1) e a aceitação dos pais sobre a necessidade do cuidado. 

“*Mas questão de agenda, de tempo né tanto nosso como também da parte das escolas, né? Porque lá eles têm uma demanda bastante grande, né? E aí a gente precisa conversar pra poder eles conseguir arrumar no calendário deles uma data em que a gente possa estar indo pra poder fazer as ações*” (P1, Norte).

## Discussão

A análise sistemática do processo de microimplementação do PROTEJA evidenciou a aceitabilidade e viabilidade ^23^ de uma estratégia nacional setorial de prevenção e atenção à obesidade infantil, com fomento à intersetorialidade em nível municipal. É importante contextualizar que o processo de implementação ocorre em um período de desmonte de políticas públicas e negação das práticas intersetoriais de gestão ^31^. Em contrapartida, seu processo de elaboração ^12^ ressalta a resiliência da institucionalidade do SUS ^31^.

Quanto ao domínio Inovação, a implementação do PROTEJA esteve alicerçada em quatro pilares: incentivo financeiro, definição de um RT municipal, suporte especializado externo e disponibilização de materiais técnicos. Este modelo replica estratégias comuns a programas direcionados para a primeira infância ^32^ e é consistente com fatores ligados à difusão de inovações, como descentralização de atividades, estrutura de comunicação organizacional, capacidade técnica dos envolvidos e presença de agentes externos ^33^. 

Diante da infraestrutura limitada, escassez de recursos humanos, financiamento restrito e afastamento geográfico de centros urbanos, é comum os municípios de pequeno porte terem baixa capacidade construída para incorporar mudanças propostas por inovações ^34^ ^,^ ^35^ ^,^ ^36^. Assim, o desenho incremental e adaptável do PROTEJA, a compatibilidade com as atribuições das SMS e a ênfase sobre a formação de rede de governança local, com olhar para o planejamento estratégico situacional ^37^, oportunizou a construção de aprendizados institucionais para o fortalecimento de práticas setoriais e intersetoriais da gestão em saúde ^15^ ^,^ ^20^ ^,^ ^38^. Entretanto, a descontinuação da estratégia, sem alternativas de continuidade, tende a reproduzir obstáculos históricos para a efetividade e sustentabilidade das estratégias de alimentação e nutrição no âmbito da saúde ^39^ ^,^ ^40^ ^,^ ^41^. 

O enfrentamento da obesidade infantil em nível local requer abordagem sistêmica, com foco nas demandas, características e infraestruturas dos territórios, envolvendo diversos setores da Administração Pública na formação de agendas convergentes e coerentes para reduzir sua prevalência ^8^ ^,^ ^28^ ^,^ ^42^ ^,^ ^43^. Assim, ao considerarmos o cenário de implementação como um sistema complexo, tem-se como premissa que o sucesso, a expansão e a sustentabilidade de inovações podem ser produzidas por múltiplos caminhos causais ^38^ ^,^ ^44^.

A aprendizagem gerada durante a implementação reduziu a percepção de Complexidade do PROTEJA pelos BNR, ao mesmo tempo em que estimulou a discricionariedade para adaptações ^15^ ^,^ ^28^. Mesmo quando o modelo de implementação proposto ^37^ não foi seguido linearmente, a execução cotidiana e sistemática das etapas permitiu a concretização da estratégia, pois muitas das ações já estavam integradas às rotinas dos serviços de saúde, educação e assistência social. Nestes casos, o PROTEJA atuou aumentando a eficiência dos processos.

Estes resultados refletem a Teoria dos Processos ^45^, na qual as partes interessadas (atores do ambiente interno e externo) estiveram envolvidas no plano organizacional (planejamento) e no plano da utilização (atividades de execução) para atingir a população. Assim, o envolvimento dos BNR, desde o planejamento até a execução das ações, implicou maior comprometimento com a mudança proposta e, como consequência da aprendizagem individual, obteve-se a aprendizagem coletiva e redução da alienação de gestores, gerando alterações organizacionais. 

Na avaliação dos 20 anos de implementação da Política Nacional de Alimentação e Nutrição (PNAN) ^40^, pontuou-se como positiva a disponibilidade e qualidade dos materiais instrutivos e dos cursos de capacitação. Porém, permanecem lacunas quanto ao alcance, efetividade e impacto destes materiais para a mudança de práticas assistenciais e de gestão. Neste contexto, o PROTEJA pode ter contribuído no preenchimento desta lacuna.

O apoio de agentes externos (ApR e ApL) dotados de características profissionais similares às dos BNR (alteridade), credibilidade e treinamento para desenvolver conexão interpessoal e transferência de conhecimento, não apenas aproximou os RTs da Inovação, mas também os instrumentalizou para operacionalizar a política na realidade local ^33^ ^,^ ^37^. Este, talvez, seja o aspecto mais importante dentre as contribuições do PROTEJA para as políticas públicas, em especial à PNAN, pois o apoio institucional nem sempre esteve presente desde o início da implementação, tendo sido gradualmente estabelecido a partir da atuação do BNR. Assim, destaca-se o papel transformador dos BNR na organização dos serviços de saúde, desde que disponham de recursos adequados de trabalho ^33^ ^,^ ^43^. 

Considerando a pactuação interfederativa no SUS, este modelo de apoio poderia ser transferido para as coordenações estaduais e regionais de alimentação e nutrição, inclusive fortalecendo e normatizando seu papel na implementação da PNAN ^40^. Diante da necessidade de ampliação das equipes técnicas, tal abordagem poderia ser viabilizada por meio da vinculação parcial dos recursos do Financiamento das Ações de Alimentação e Nutrição (FAN) direcionados aos estados.

A inclusão de ações de governança como essenciais (p.ex.: formação de GTI e formalização de planos de ação), ao invés de um procedimento implícito de gestão, foi um diferencial do PROTEJA. Contudo, a adoção de modelo horizontalizado e participativo de governança requer mudanças estruturais das instituições ^46^. Barreiras como comunicação intersetorial limitada e indisponibilidade de tempo das equipes externas impactaram o funcionamento dos GTIs. Ainda, a incompreensão técnica ou política da estratégia e a falta de autonomia dos gestores dificultaram a articulação intersetorial ^33^ ^,^ ^43^ ^,^ ^46^. 

As disputas de agenda pública no Ambiente Externo estendem-se ao Ambiente Interno. A concorrência de agendas setoriais e o não reconhecimento da obesidade infantil como problema público por BME e BAE podem minimizar sua Prioridade Relativa. Este fato é sugestivo de alienação sobre o cenário epidemiológico ou a presença de conflitos de interesses ^47^. Tal contexto destaca a importância da ação política das equipes técnicas dos municípios, a fim de impulsionar agendas ligadas à má-nutrição, especialmente em intervenções que demandem cooperação intersetorial.

O PROTEJA atrelou os repasses financeiros ao cumprimento de metas, impulsionando sua Prioridade Relativa ^33^. No entanto, esse formato induziu os municípios a priorizarem ações setoriais, em detrimento das ações estruturantes. A fim de corrigir tal distorção, as oficinas e atividades de apoio técnico buscaram sensibilizar os RTs quanto à equiparação das prioridades entre os repasses financeiros e os objetivos da estratégia, reforçando sua compatibilidade com as rotinas da SMS, buscando maximizar a probabilidade de sucesso ^33^ ^,^ ^47^.

Paradoxalmente, embora os BNR valorizassem os indicadores vinculados aos repasses financeiros, a execução orçamentária foi uma das principais barreiras para a implementação, diante da baixa proficiência em sua utilização. Nestes municípios, infere-se que tal dificuldade esteja ligada ao conhecimento insuficiente sobre os fluxos da administração pública para a execução orçamentária devido às poucas oportunidades de acessarem fontes específicas de financiamento, como o FAN, dado que municípios com menos de 30 mil habitantes não têm direito a esta fonte de financiamento. Uma vez que dificuldades de acesso aos recursos diminuem o engajamento dos BNR com a inovação ^33^ ^,^ ^48^, este tema foi trabalhado de modo específico e transversal nas oficinas. 

Este foi o primeiro estudo nacional a utilizar o CFIR para avaliar a implementação de uma política de alimentação e nutrição. Sua adoção como base conceitual para a elaboração do roteiro de entrevistas e codificação dos dados garantiu um referencial robusto para identificar, categorizar e interpretar os principais determinantes da implementação do PROTEJA, especialmente pela distinção entre Ambiente Interno e Ambiente Externo, dada a natureza intersetorial da estratégia. Outro aspecto positivo do estudo está na diversidade geográfica, socioeconômica e de performance da amostra, possibilitando a transferência direta de conhecimento para a formulação de políticas públicas.

Ainda assim, algumas limitações devem ser reconhecidas. A exclusão do domínio “características dos indivíduos”, embora justificada, pode restringir a compreensão das subjetividades que influenciam a implementação, como valores, crenças e agência dos profissionais. Já a codificação dedutiva, baseada em categorias pré-estabelecidas, pode ter limitado a emergência de significações relevantes para o processo de implementação. Por fim, a utilização de frequências de códigos como recurso descritivo requer interpretação cautelosa, dado que a recorrência de menções não é necessariamente indicativa de maior ou menor relevância da categoria/domínio.

## Conclusão

Ao articular diretrizes técnicas, apoio institucional e incentivos financeiros, viabilizou-se a implementação de uma estratégia intersetorial nacional de prevenção da obesidade infantil. A análise orientada pelo CFIR evidenciou como a interação entre elementos técnicos (instrumentos de planejamento e metas), organizacionais (estrutura e redes intra e intersetoriais) e humanos (capacidades instaladas e trajetórias de aprendizado) facilitaram ou dificultaram a materialização da política. Identificou-se a importância de estruturas locais de governança intersetorial para o compartilhamento de agendas, recursos e responsabilidades, a fim de aumentar a coesão política sobre um problema público. Para assegurar a sustentabilidade das políticas de alimentação e nutrição, há necessidade de estabelecer estratégias de valorização profissional e institucionalizar e qualificar mecanismos de apoio técnico aos BNR para a superação de barreiras ligadas à articulação intersetorial, utilização de recursos financeiros e na ação política junto aos BME e BAE para promoção de agendas públicas.

Mediado pela PNAN, tendo em vista o conjunto de ações essenciais e complementares do PROTEJA, as lições aprendidas no processo de implementação podem contribuir para a integração do Sistema Único de Saúde (SUS), Sistema Nacional de Segurança Alimentar e Nutricional (SISAN), Sistema Único de Assistência Social (SUAS) e o Programa Nacional de Alimentação Escolar (PNAE) para acabar com todas as formas de má-nutrição.

## Data Availability

Os dados de pesquisa estão disponíveis mediante solicitação à autora de correspondência.
